# Supplementary material for: Apoptosis-mediated ADAM10 activation removes a mucin barrier promoting T cell efferocytosis
Source: Nat Commun. 2024 Jan 15;15:541. doi: 10.1038/s41467-023-44619-8 (PMC10789802; doi:10.1038/s41467-023-44619-8)
Supplement: Supplementary file 1 — Supplementary Information [file 41467_2023_44619_MOESM1_ESM.docx]

**Supplementary Figures (11 figures) for:**

**Apoptosis-mediated ADAM10 activation removes a mucin barrier promoting T cell**

**Efferocytosis**

Linnea Drexhage et al

**Corresponding authors**: Quentin Sattentau: [quentin.sattentau@path.ox.ac.uk,](mailto:quentin.sattentau@path.ox.ac.uk) Niloofar Karaji: niloofar.karaji@gmail.com


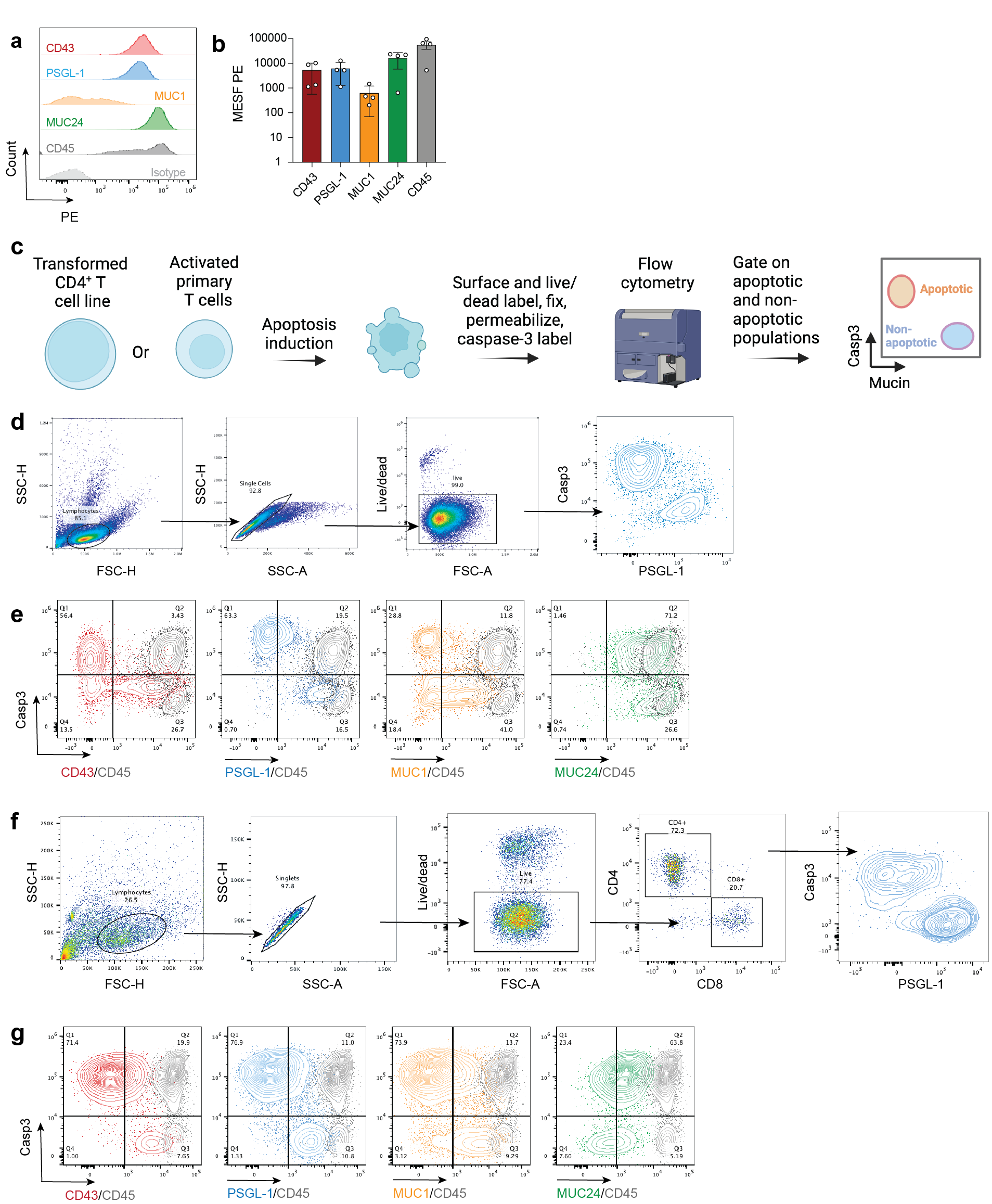


**Supplementary Fig. 1. Analysis of mucin expression on the surface of apoptotic T cells.**

**a**, Relative cell surface mucin expression on CEM analyzed by flow cytometry.

**b**, Number of mucin molecules estimated per CEM cell using Molecules of Equivalent Soluble Fluorochrome (MESF) quantification, *n* = 4 independent experiments.

**c**, Workflow for apoptosis induction, detection and mucin expression on healthy and apoptotic T cells (created with Biorender.com).

**d**, Flow cytometry gating strategy for analysis of mucin expression on apoptotic and non-apoptotic CEM; side-scatter = SSC, forward scatter = FSC.

**e**, Dot plot of activated caspase-3 (Casp3) vs mucin expression on the surface of staurosporine-treated CEM, all mucins compared to CD45. Data comparing Casp3 with mucin expression (in this case PSGL1) shown in the right-hand end panel of (d). These data were gated as in (e) to provide % positive populations of apoptosis (y-axis) against mucin expression (x-axis). This was used to generate quantified data for main figures 1a,b,d,e; 2a,b; 3b,c and e,f; supplementary figures 2c-h; 3a-d; 4a-c and f-i; 5b; 7d,e; 9a-c and f.

**f**, Flow cytometry gating strategy for analysis of mucin expression on apoptotic and non-apoptotic primary T cells gated on either CD4^+^ or CD8^+^ populations and then analyzed for apoptosis induction by activated Casp3 labelling (*y*-axis) against mucin expression (*x*-axis) as in the right-hand end panel.

**g**, Data were gated as in (f) to provide % positive populations of apoptosis (*y*-axis) against mucin expression (*x*-axis). This was used to generate quantified data for main figures 1c and supplementary figures 2a,b,i; 4d,e and 5e.

All error bars represent ± 1 SD around the mean.


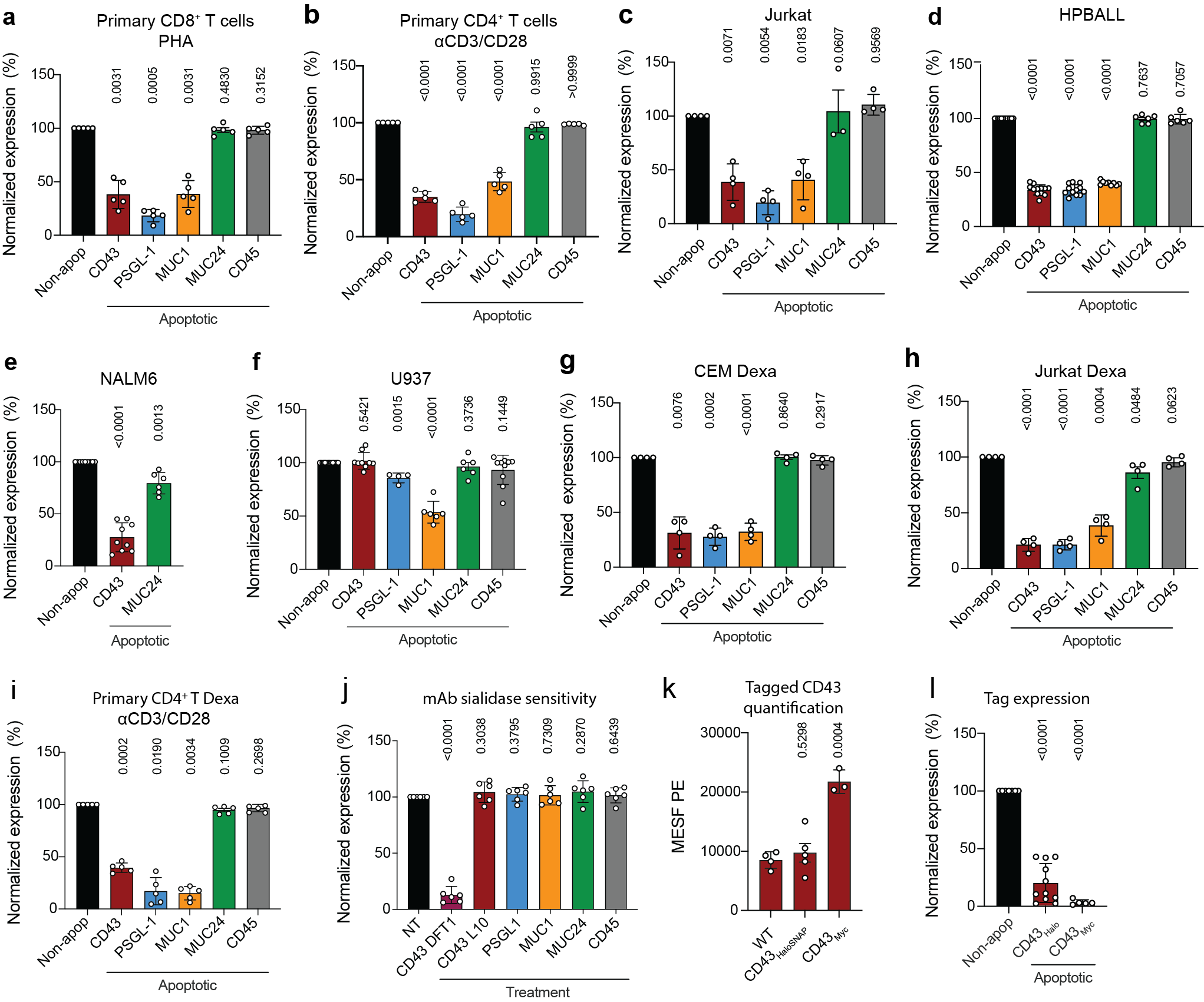


**Supplementary Fig. 2. Mucin expression on apoptotic immune cells**.

**a**, Mucin expression on primary CD8^+^ T cells activated with PHA + IL-2 and induced for apoptosis with staurosporine; *n* = 5 independent donors.

**b**, Mucin expression on primary CD4^+^ T cells activated with CD3/CD28 beads + IL-2 and induced for apoptosis with staurosporine; *n* = 5 independent donors.

**c**, Mucin expression on Jurkat T cells induced for apoptosis with staurosporine; *n* = 4 independent experiments.

**d**, mucin expression on HPBALL induced for apoptosis with staurosporine; *n* = 6-14 independent experiments.

**e**, Mucin expression on NALM6 induced for apoptosis with staurosporine; *n* = 6–9 independent experiments.

**f**, Mucin expression on U937 induced for apoptosis with staurosporine; *n* = 6–10 independent experiments.

**g**, Mucin expression on CEM induced for apoptosis using dexamethasone (Dexa); *n* = 4 independent experiments.

**h**, Mucin expression on Jurkat induced for apoptosis using Dexa; *n* = 4 independent experiments.

**i**, Mucin expression on primary CD4^+^ T cells activated with CD3/CD28 + IL-2 and induced for apoptosis with Dexa; *n* = 5 independent donors.

**j**, Mucin-specific antibody binding to sialidase-treated CEM, with data normalized to mucin expression on non-sialidase-treated (NT) cells set at 100% and shown as a single bar; *n* = 3 independent experiments. Panels a-j, one-way ANOVA with Dunnett’s post-hoc test.

**k**, Relative CD43_Myc_ and CD43_Halo_ expression levels determined by MESF on CEM-CD43_KO_ cells stably transfected with an N-terminal Halo and C-terminal SNAP tag (CD43_Halo/SNAP_) or N-terminal Myc tag (CD43_Myc_); *n* = 3–5 independent experiments. Unpaired *t* tests.

**l**, Loss of CD43_Myc_ and CD43_Halo_ labels during apoptosis relative to non-apoptotic counterparts, both normalized to 100% shown as a single bar; *n* = 5–12 independent experiments. Log_10_-transformed, one-way ANOVA with Dunnett’s post-hoc test.

In panels a-j and l, non-apoptotic cells (Non-apop) were normalized to 100% and shown as a single bar. All error bars represent ± 1 SD around the mean.


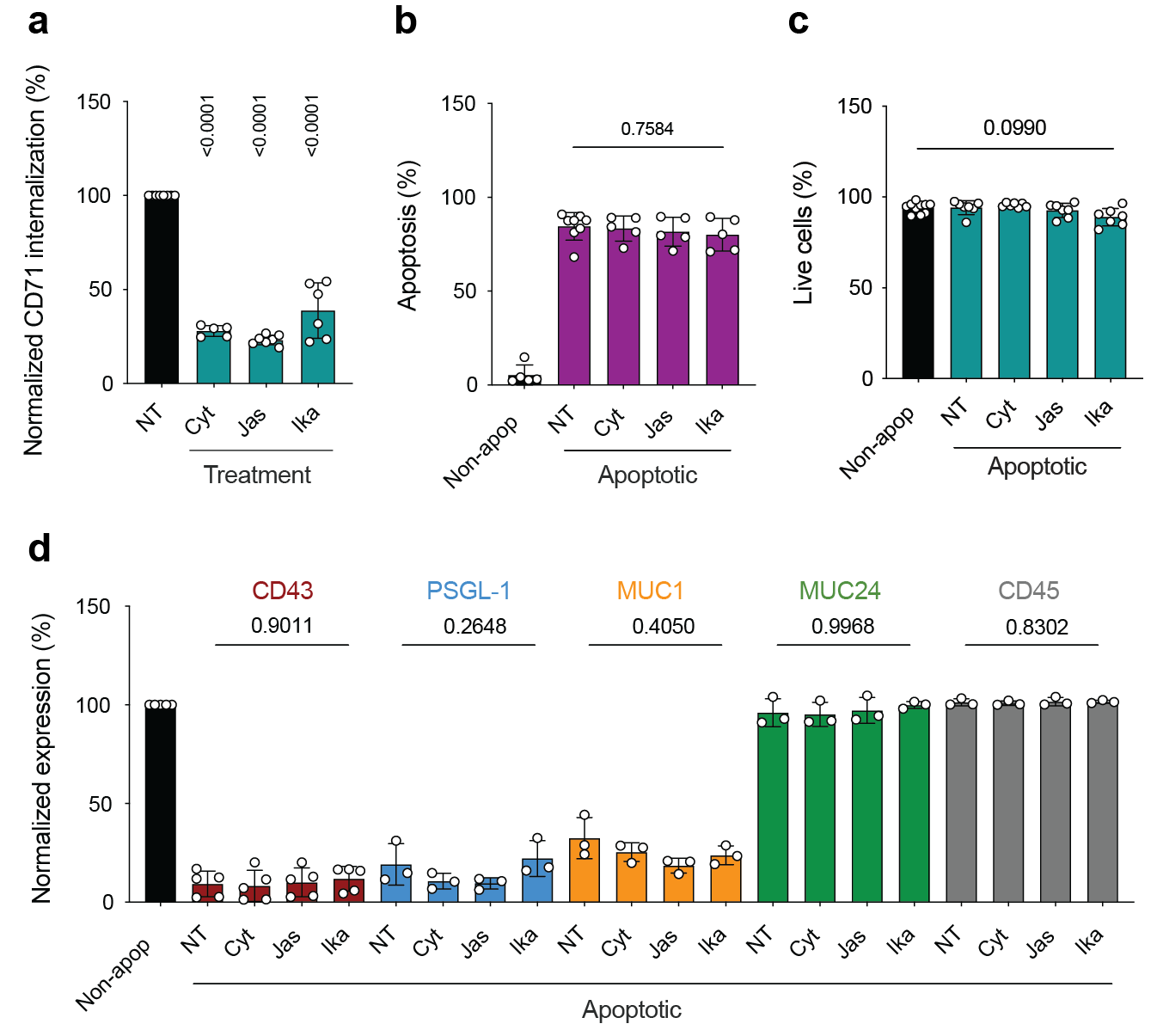


**Supplementary Fig. 3. Inhibition of actin remodeling and endocytosis does not prevent mucin loss**.

**a**, Effect of actin remodeling inhibitors cytochalasin-D (Cyt), Jasplakinolide (Jas) and endocytosis inhibitor Ikarugamycin (Ika) on antibody-mediated CD71 internalization, non-treated (NT) cells normalized to 100%; *n* = 5–8 independent experiments. One-way ANOVA of log_10_-transformed data with Dunnett’s post-hoc test.

**b**, Effect of inhibitors on staurospaurine-induced apoptosis assayed by activated caspase-3 labeling, *n* = 5–8 independent experiments. One-way ANOVA.

**c**, Lack of inhibitor-mediated cytotoxicity, live (apoptotic and non-apoptotic) cells gated using live/dead stain, *n* = 5–7 independent experiments. One-way ANOVA.

**d**, Lack of effect of inhibitors on mucin loss during apoptosis, *n* = 3–5 independent experiments. One-way ANOVA of log_10_-transformed data.

In panels c,d, non-apoptotic cells (Non-apop) were normalized to 100% and shown as a single bar. All error bars represent ± 1 SD around the mean.


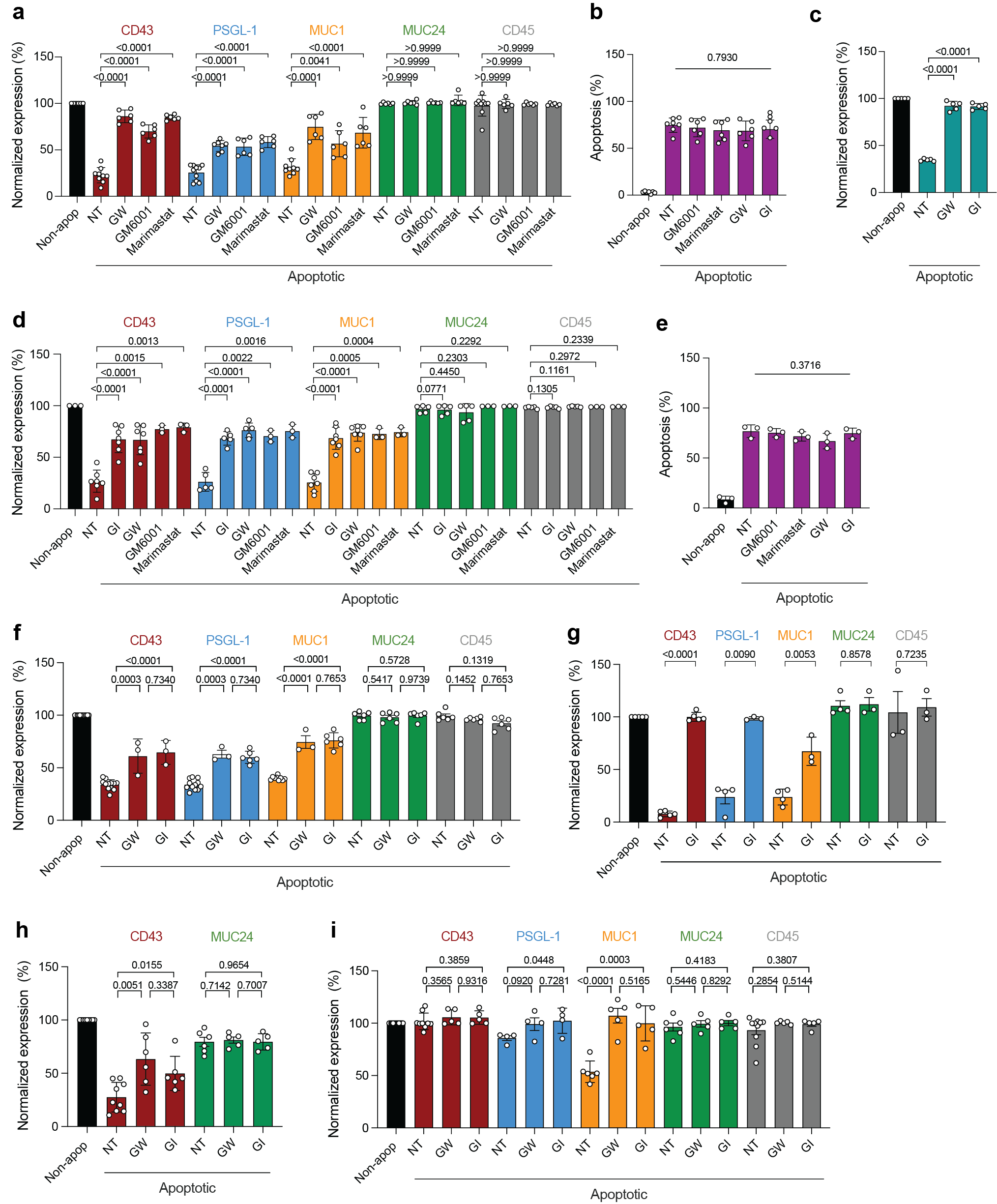


**Supplementary Fig. 4. Metalloprotease inhibitors reduce mucin shedding**.

**a**, Metalloprotease inhibitors were added to staurospaurine-treated CEM and cells labelled for activated caspase-3 and mucin expression, NT = non-treated; *n* = 6–11 independent experiments. One-way ANOVA of log_10_-transformed data with Dunnett’s post-hoc test.

**b**, Effect of metalloprotease inhibitors on staurosporine-induced CEM apoptosis; *n* = 6–7 independent experiments. One-way ANOVA.

**c**, CD46 expression on staurosporine-treated CEM, pre-treated or NT with metalloprotease inhibitors, *n* = 5 independent experiments. One-way ANOVA of log_10_-transformed data with unpaired *t* tests.

**d**, Mucin expression on staurospaurine-treated primary CD4^+^ T cells pre-treated or untreated (NT) with metalloprotease inhibitors, *n* = 3–9 independent donors. One-way ANOVA of log_10_-transformed data with Dunnett’s post-hoc test.

**e**, Effect of metalloprotease inhibitors on staurosporine-induced primary T cell apoptosis determined by activated caspase-3 labeling; *n* = 3 independent experiments. One-way ANOVA.

**f**, Mucin expression on staurosporine-treated HPBALL, pre-treated or untreated (NT) with metalloprotease inhibitors GW or GI, *n* = 3–12 independent experiments.

**g**, Mucin expression on staurosporine-treated Jurkat, pre-treated or NT with ADAM10 inhibitor GI, non-apoptotic (Non-apop) cells normalized to 100% shown as a single bar; *n* = 3–5 independent experiments.

**h**, Mucin expression on staurosporine-treated NALM6, pre-treated or not treated (NT) with metalloprotease inhibitors GW or GI, *n* = 5–9 independent experiments.

**i**, Mucin expression on staurosporine-treated U937, pre-treated or untreated (NT) with ADAM10 inhibitor GI, *n* = 4–8 independent experiments.

Panels f-i, Log_10_-transformed unpaired *t* tests. Panels a,c,d and f-i, non-apoptotic cells (Non-apop) were normalized to 100% and shown as a single bar. All error bars represent ± 1 SD around the mean.


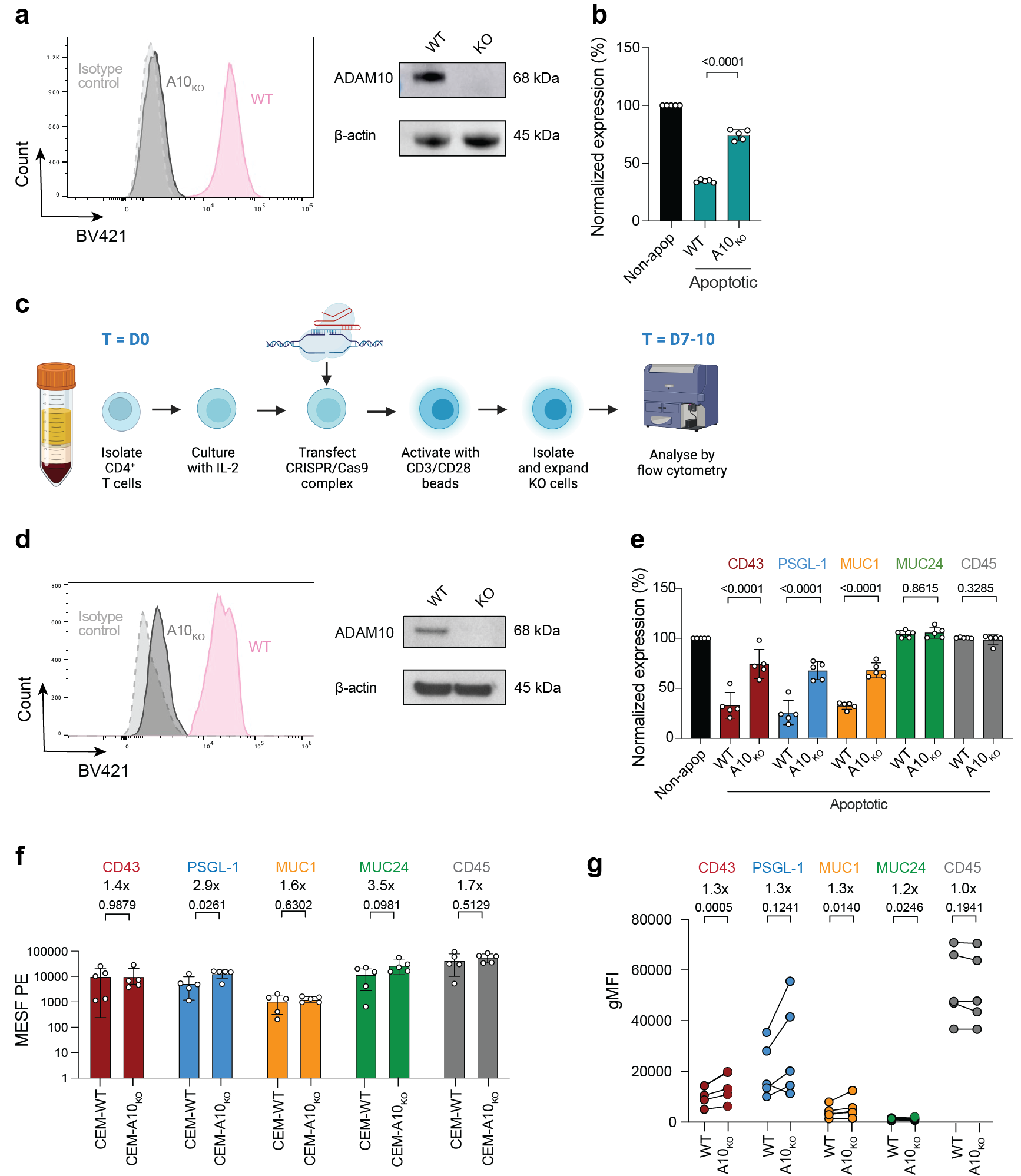


**Supplementary Fig. 5. CRISPR-Cas9 knock out of ADAM10 inhibits mucin shedding.**

**a**, Analysis of cell surface ADAM10 expression, CEM WT and CRISPR-Cas9 KO line ADAM10_KO_ = A10_KO_, dotted grey line = isotype control, and western blot of CEM lysate for A10_KO_ compared to WT CEM, β-actin = loading control, molecular weight (kDa) of band to right of blot.

**b**, Cleavage of CD46 in apoptotic CEM WT and A10_KO_, non-apoptotic cells (Non-apop) normalized to 100% shown as a single bar, *n* = 5 independent experiments. Log_10_ transformed data with unpaired *t* test.

**c**, Workflow for ADAM10 CRISPR-Cas9 KO in primary CD4^+^ T cells (created with Biorender.com).

**d**, Flow cytometric cell surface and western blot analysis of CRISPR-Cas9 A10_KO_ primary CD4^+^ T cells treated with staurosporine and analyzed by flow cytometry, and western blot of T cell lysate for A10_KO_ compared to WT T cells, β-actin = loading control, molecular weight (kDa) of band to right of blot.

**e**, Staurosporine-treated WT and A10_KO_ primary T cells analyzed for mucin expression, non-apoptotic cells (Non-apop) normalized to 100% and represented as a single bar for all groups; *n* = 5 independent experiments. Log_10_-transformed data with unpaired *t* tests.

**f**, MESF analysis of mucin expression on healthy CEM WT and A10_KO_, numbers above bars reflect fold-increase in expression; *n* = 5 independent experiments. Unpaired *t* tests.

**g**, Flow cytometric analysis of mucin expression on healthy primary CD4^+^ T cells expressed as geometric mean fluorescence (gMFI), numbers above bars reflect fold-increase in expression; *n* = 5 independent donors. Paired *t* tests.

All error bars represent ± 1 SD around the mean.


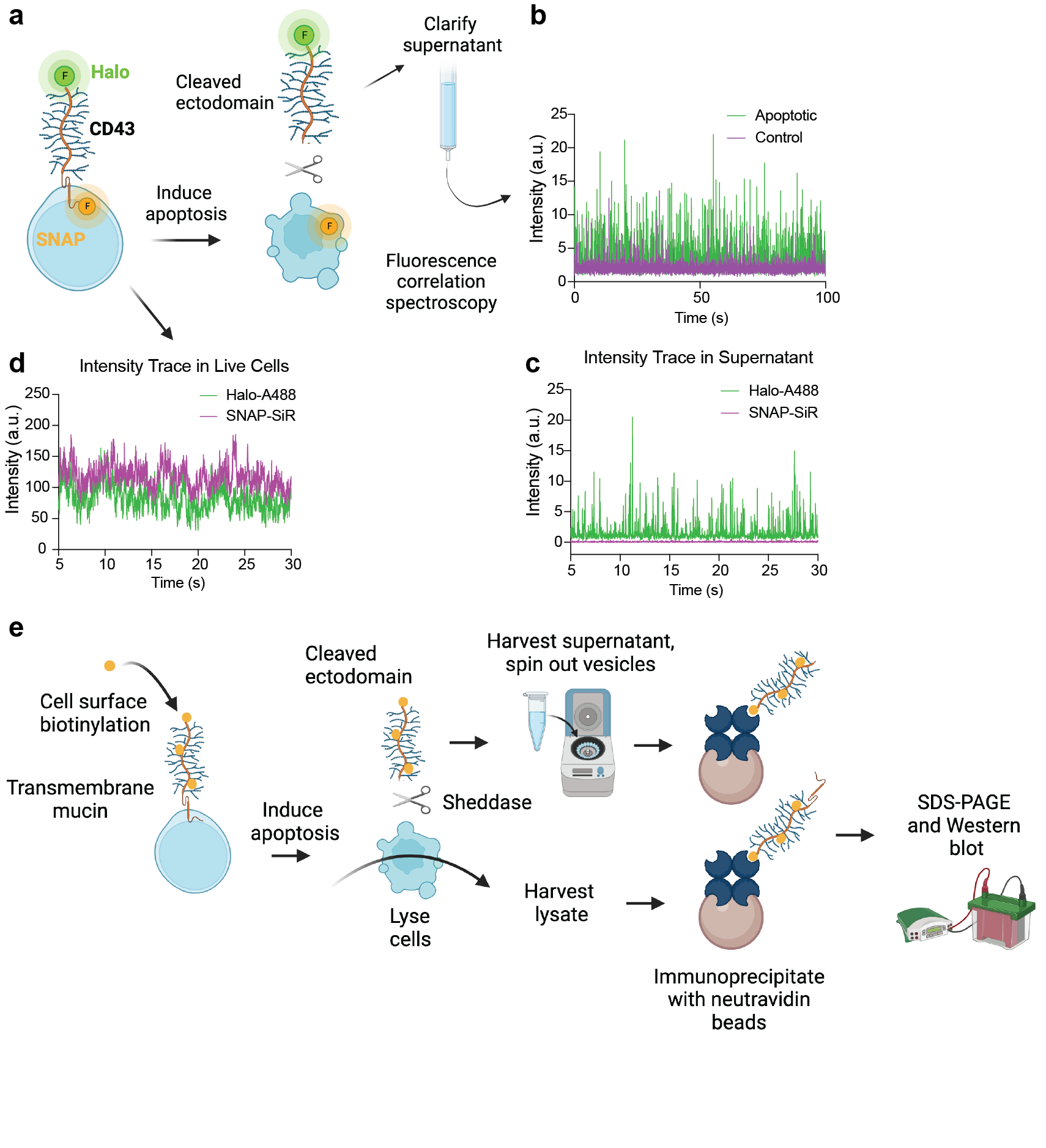


**Supplementary Fig. 6. Mucin ectodomains are shed from apoptotic cells.**

**a,** Workflow for treatment of CEMHalo/SNAP cells and Fluorescence Correlation Spectroscopy (FCS) analysis.

**b,** Time-resolved fluorescent single-molecule Halo-A488 peaks observed in processed supernatant from apoptotic compared to non-apoptotic CEM.

**c,** Time-resolved intensity trace in supernatant of apoptotic CEM of Halo-A488 and SNAP-SIR labels.

**d,** Time-resolved intensity trace of Halo-A488 and SNAP-SIR fluorescence in healthy CEMHalo/SNAP cells.

**e,** Workflow for cell surface biotinylation and western blot analysis of cell-associated and soluble ectodomain mucin expression (panels a and e created with Biorender.com).

**
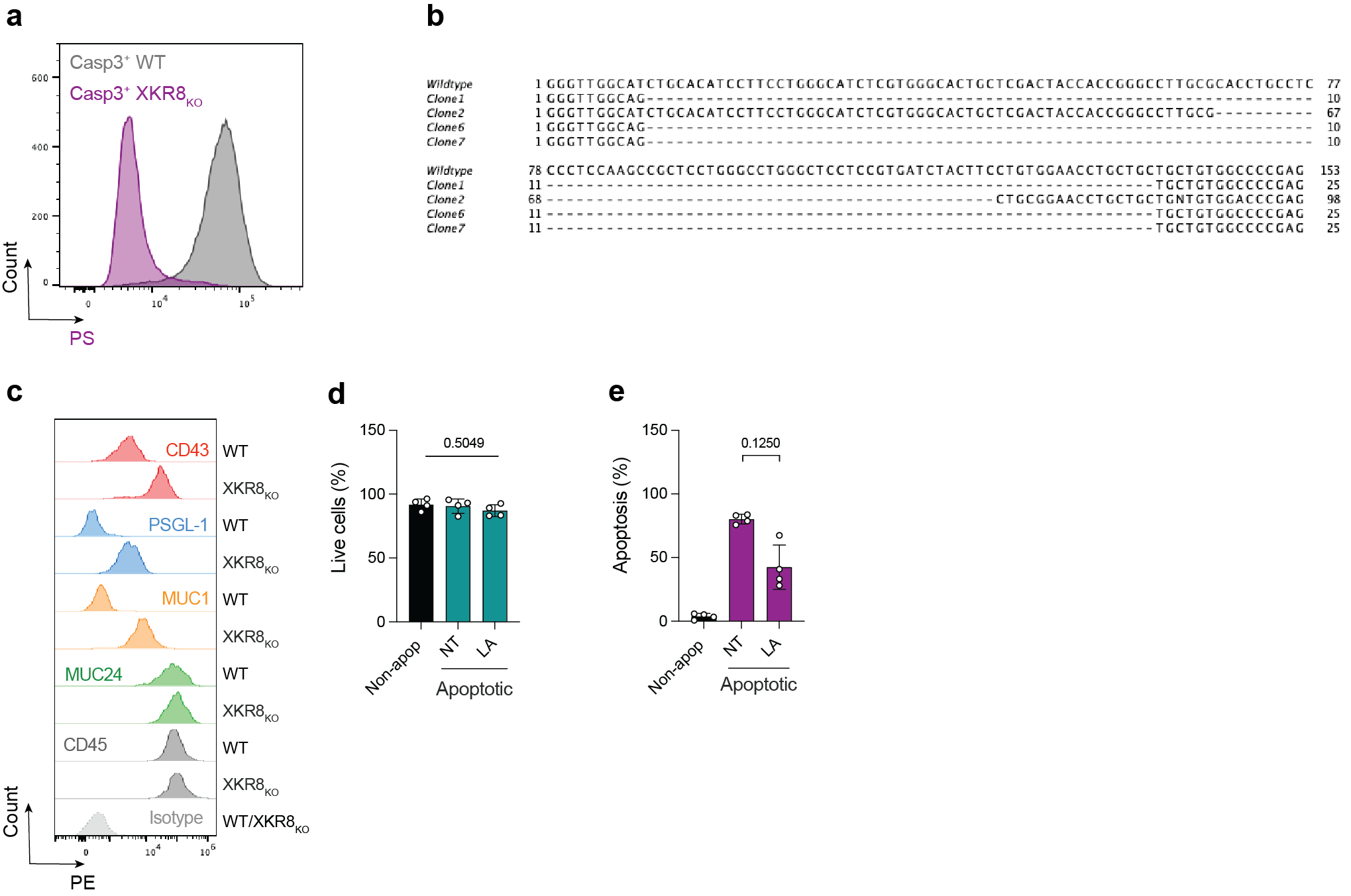
**

**Supplementary Fig. 7. XKR8_KO_ and inhibition of PS-ADAM10 interaction.**

**a**, Apoptosis induction in XKR8_KO_ CEM clone does not flip PS, assayed by annexin-V labelling of caspase-3-positive apoptotic cells.

**b**, Sequence of XKR8_KO_ PCR products to confirm deletions in Exon 3 of 4 knockout clones compared to WT CEM.

**c**, Flow cytometric analysis showing XKR8_KO_ in apoptotic CEM inhibits loss of CD43, PSGL-1 and MUC1.

**d**, LA is non-cytotoxic to apoptotic CEM cells; *n* = 4 independent experiments. One-way ANOVA.

**e**, LA does not interfere with staurosporine-induced apoptosis in CEM cells, apoptosis quantified by detection of activated caspase-3 in fixed, permeabilized cells. *n* = 4 independent experiments. Wilcoxon signed-rank test.

All error bars represent ± 1 SD around the mean.

**
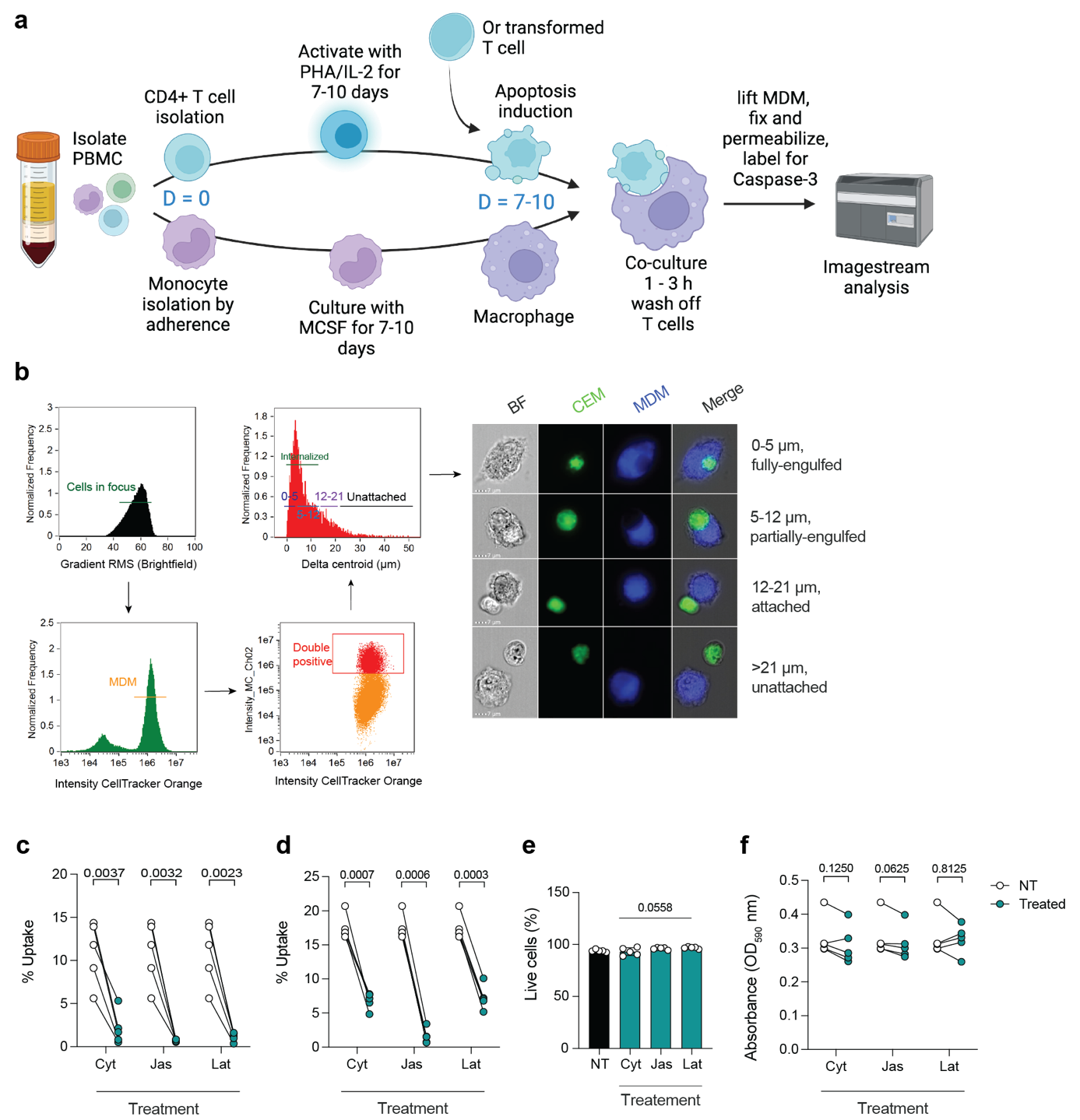
**

**Supplementary Fig. 8. Imagestream-based efferocytosis assay.**

**a**, Processing workflow for peripheral blood mononuclear cells (PBMC) to obtain activated CD4^+^ T cells and monocyte-derived macrophages (MDM), coculture and Imagestream analysis of T cell uptake (created with Biorender.com).

**b**, Imagestream gating strategy comprising selection of in-focus cells, selection of MDM, gating on double-positives and incorporation of the delta-centroid function with example images, bright-field = BF. Scale bar = 7 μm, distances are in μm.

**c**, Pre-treatment (green circles) or not (open circles) of MDM with actin remodelling inhibitors cytochalasin-D (Cyt), jasplakinolide (Jas) or latrunculin (Lat) prior to coculture with apoptotic CEM, and Imagestream analysis for T cell uptake; *n* = 5 independent experiments. Paired *t* tests.

**d**, Pre-treatment or not of MDM with actin remodelling inhibitors prior to coculture with fluorescent beads and flow cytometry analysis for bead uptake; *n* = 5 independent experiments. Paired *t* tests of log_10_-transformed data.

**e**, Flow cytometric analysis of live/dead viability labelling of untreated (NT) and actin remodelling inhibitor-treated MDM. *n* = 5 independent experiments. One-way ANOVA.

**f**, MTT assay for metabolic function of untreated (NT) and actin remodelling inhibitor-treated MDM. *n* = 5 independent experiments. Wilcoxon rank-signed tests.

All error bars represent ± 1 SD around the mean.


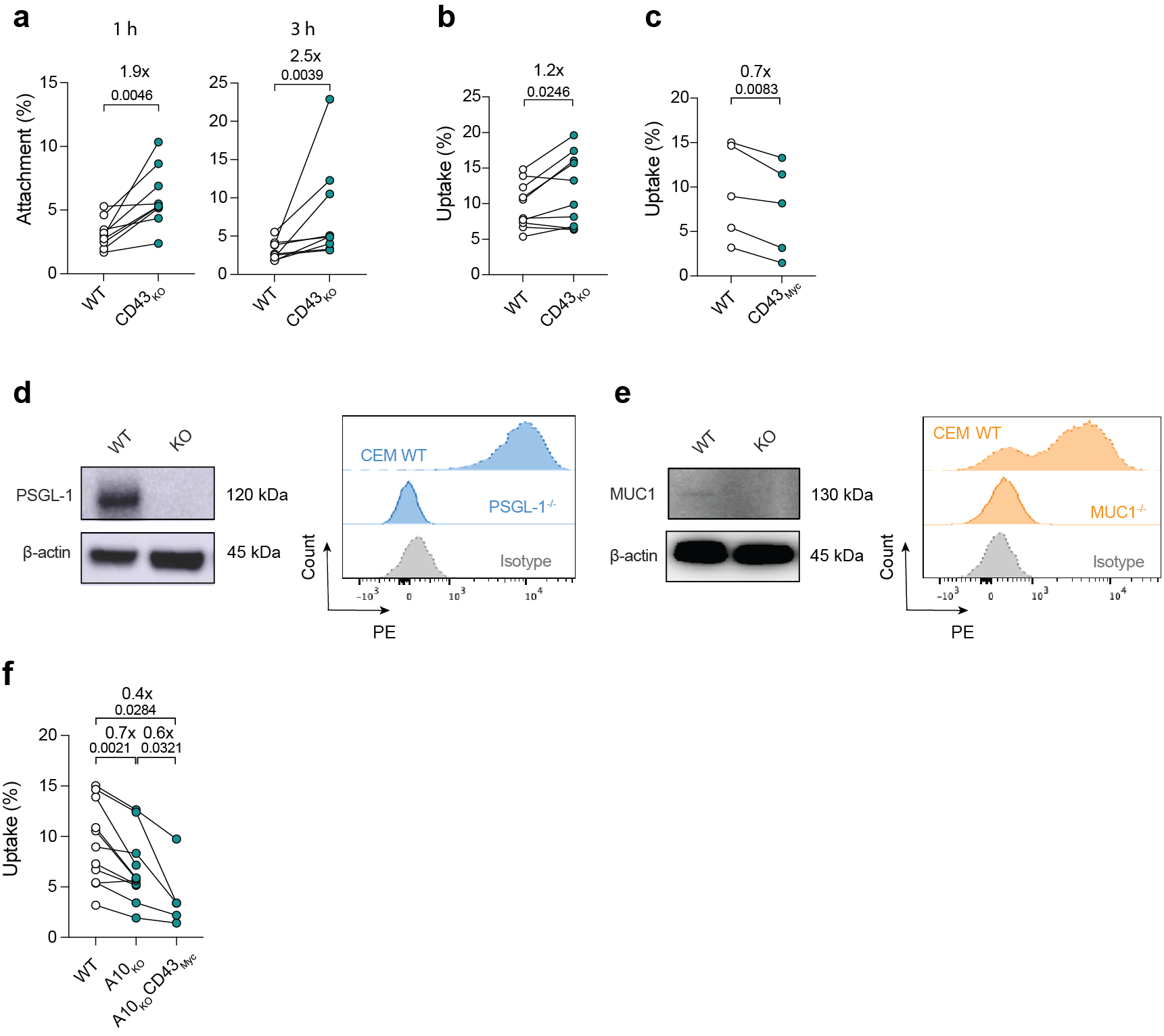


**Supplementary Fig. 9. Mucin loss enhances attachment to, and uptake of, apoptotic T cells.**

**a,** Imagestream quantification of apoptotic WT (open circles) and CD43KO (green circles) CEM attachment to MDM at T = 1 and 3 h; *n* = 6–9 independent experiments. Left panel, paired *t* test; right panel, Wilcoxon rank-signed test.

**b,** Imagestream quantification of apoptotic CEM cell uptake by MDM after 3 h coculture comparing WT (open circles) and CD43KO (green circles); *n* = 10 independent experiments. Paired *t* test.

**c**, Imagestream quantification of apoptotic CEM cell uptake by MDM after 3 h coculture comparing WT (open circles) and CD43KO overexpressing Myc-tagged CD43 (CD43Myc, green circles). *n* = 5 independent experiments. Paired *t* test.

**d**, CRISPR-Cas9 KO of PSGL-1, western blot with β-actin loading control with molecular weight (kDa) of band to right of blot, and cell surface flow cytometric analysis.

**e,** CRISPR-Cas9 KO of MUC1, western blot with β-actin loading control with molecular weight (kDa) of band to right of blot, and cell surface flow cytometric analysis.

**f,** Imagestream analysis of apoptotic CEM cell uptake by MDM after 3 h coculture comparing CEM_WT_, ADAM10KO (A10KO) and A10KO overexpressing Myc-tagged CD43 (A10KOCD43Myc); *n* = 5–10 independent experiments. Log_10_-transformed data with paired *t* tests.

All error bars represent ± 1 SD around the mean.


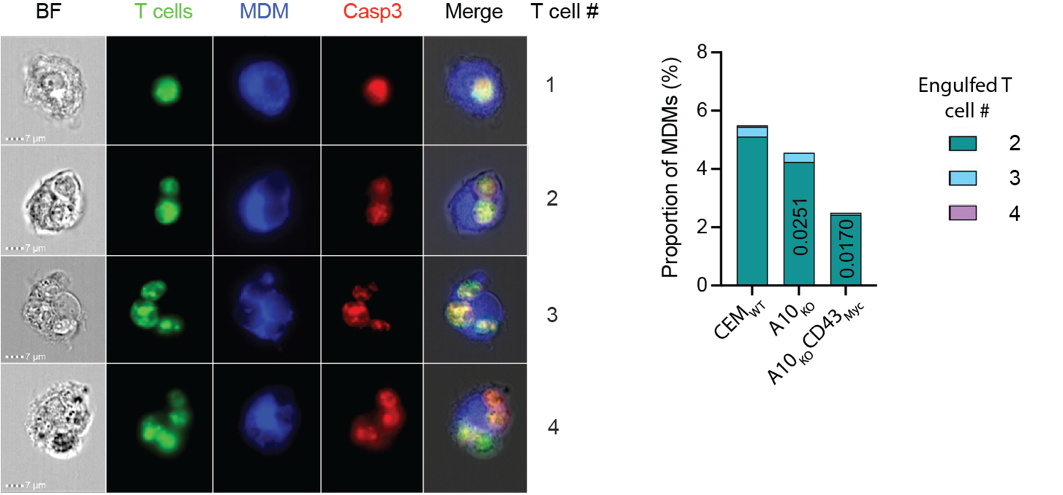


**Supplementary Fig. 10. MDM engulf multiple apoptotic CEM.**

Staurosporine-treated CEM cells were washed, cocultured with MDM for 1 h, washed in cold PBS/EDTA, lifted, fixed, permeabilized and labelled for CD3 (T cells) and caspase-3 (Casp3). Numbers of T cells in 1000 independent images double-positive for MDM and T cells were manually counted. MDM containing single T cells were excluded from the quantification to prioritize display of multiple uptake events. WT CEM (CEMWT) were compared with CEM A10KO and CEM A10KOCD43Myc, unpaired *t* tests.


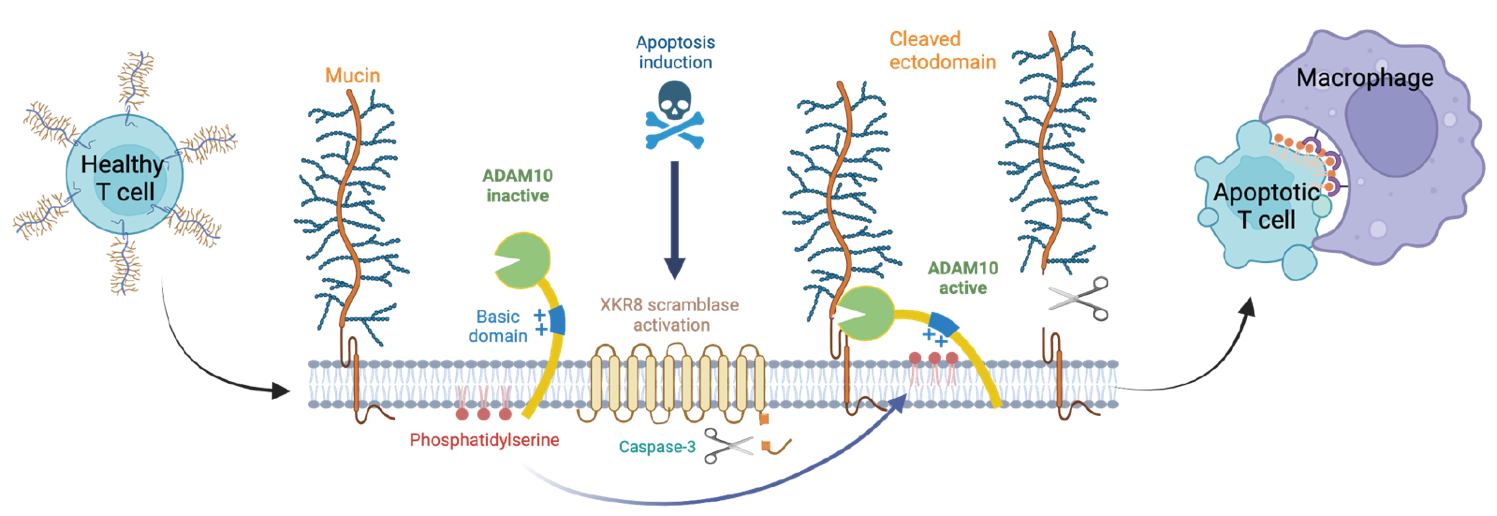


#
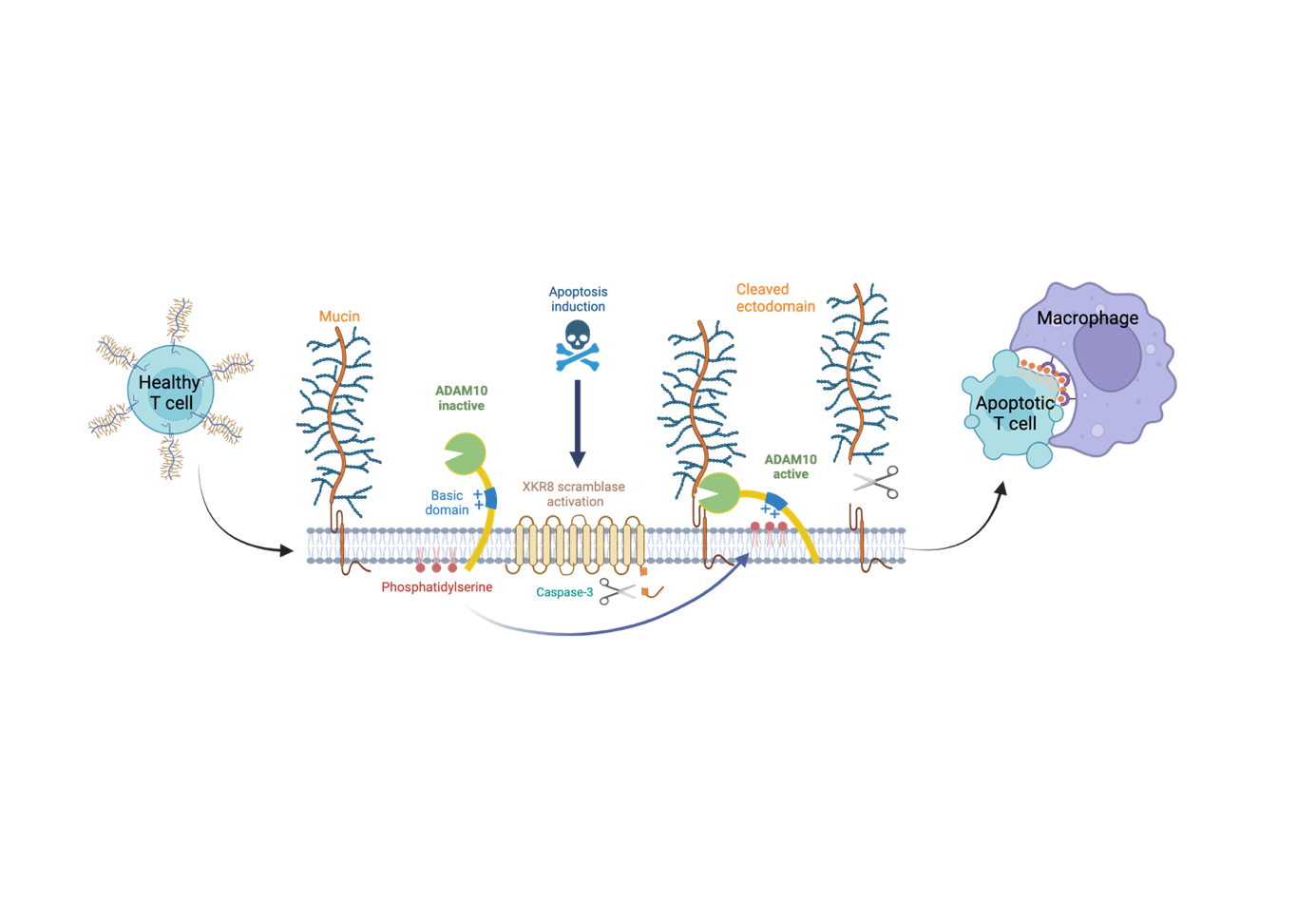


**Supplementary Fig. 11. Model for mucin loss facilitating T cell efferocytosis by macrophages**.

Healthy T cells express a high density of extended transmembrane mucins in their glycocalyx. Activation of apoptosis leads to flipping of phosphatidylserine (PS) most likely via caspase-3 activation of the scramblase XKR8, which in turn activates ADAM10 sheddase activity for selected mucins. Mucin cleavage depletes the glycocalyx biophysical barrier and better exposes membrane-proximal eat-me signals such as PS, which are recognized by cognate phagocytic receptors leading to efficient efferocytosis (created with Biorender.com).
