# Supplementary material for: Apoptosis-mediated ADAM10 activation removes a mucin barrier promoting T cell efferocytosis
Source: Nat Commun. 2024 Jan 15;15:541. doi: 10.1038/s41467-023-44619-8 (PMC10789802; doi:10.1038/s41467-023-44619-8)
Supplement: Supplementary file 3 — Reporting Summary [file 41467_2023_44619_MOESM3_ESM.pdf]

nature portfolio

Corresponding author(s): Quentin Sattentau and Niloofar Karaji

Last updated by author(s): 2023/08/18

Reporting Summary

Nature Portfolio wishes to improve the reproducibility of the work that we publish. This form provides structure for consistency and transparency in reporting. For further information on Nature Portfolio policies, see our Editorial Policies and the Editorial Policy Checklist.

Statistics

For all statistical analyses, confirm that the following items are present in the figure legend, table legend, main text, or Methods section.

n/a	Confirmed	
☐	☒	The exact sample size (n) for each experimental group/condition, given as a discrete number and unit of measurement
☐	☒	A statement on whether measurements were taken from distinct samples or whether the same sample was measured repeatedly
☐	☒	The statistical test(s) used AND whether they are one- or two-sided<br>*Only common tests should be described solely by name; describe more complex techniques in the Methods section.*
☒	☐	A description of all covariates tested
☐	☒	A description of any assumptions or corrections, such as tests of normality and adjustment for multiple comparisons
☐	☒	A full description of the statistical parameters including central tendency (e.g. means) or other basic estimates (e.g. regression coefficient) AND variation (e.g. standard deviation) or associated estimates of uncertainty (e.g. confidence intervals)
☐	☒	For null hypothesis testing, the test statistic (e.g. F, t, r) with confidence intervals, effect sizes, degrees of freedom and P value noted<br>*Give P values as exact values whenever suitable.*
☒	☐	For Bayesian analysis, information on the choice of priors and Markov chain Monte Carlo settings
☒	☐	For hierarchical and complex designs, identification of the appropriate level for tests and full reporting of outcomes
☒	☐	Estimates of effect sizes (e.g. Cohen's d, Pearson's r), indicating how they were calculated

Our web collection on statistics for biologists contains articles on many of the points above.

Software and code

Policy information about availability of computer code

Data collection	Flow cytometry data acquired using CytExpert V2.5 (Beckman Coulter)<br>Imagestream images were acquired with INSPIRE V4 software.
Data analysis	Flow cytometry data were processed using the FlowJo-V10 software (FlowJo, LLC), and molecules of equivalent soluble fluorochrome data were analyzed using Bangs Laboratories QuickCal v 3.0.<br>Blots were density quantified using LI-COR ImageStudio V5.2.5 software.<br>Imagestream images were analyzed using IDEAS 6.2.

For manuscripts utilizing custom algorithms or software that are central to the research but not yet described in published literature, software must be made available to editors and reviewers. We strongly encourage code deposition in a community repository (e.g. GitHub). See the Nature Portfolio guidelines for submitting code & software for further information.

Data

Policy information about availability of data

All manuscripts must include a data availability statement. This statement should provide the following information, where applicable:

- Accession codes, unique identifiers, or web links for publicly available datasets
- A description of any restrictions on data availability
- For clinical datasets or third party data, please ensure that the statement adheres to our policy

Custom code was not used in this study. The methods, reagents, targets, and verified hits from this study are contained in the manuscript. Unprocessed data for all figures are provided as source data files with the manuscript. Sequence databases used are listed in the methods section and here: https://www.ensembl.org/Homo_sapiens/Gene/Summary?db=core;g=ENSG00000110876; https://www.ensembl.org/Homo_sapiens/Gene/Summary?db=core;g=ENSG00000185499; NP_001101.1, https://www.ncbi.nlm.nih.gov/protein/NP_001101.1; https://www.ensembl.org/Homo_sapiens/Gene/Summary?db=core;g=ENSG00000158156

Research involving human participants, their data, or biological material

Policy information about studies with human participants or human data. See also policy information about sex, gender (identity/presentation), and sexual orientation and race, ethnicity and racism.

Reporting on sex and gender	All human samples were anonymized at source
Reporting on race, ethnicity, or other socially relevant groupings	all human samples were anonymized at source
Population characteristics	all human samples were anonymized at source
Recruitment	Blood products were sourced anonymously from the local National Health Service (NHS) blood bank
Ethics oversight	Leukocyte cones from anonymous healthy donors were provided by the National Health Service UK (NHS) Blood and Transplant services (Oxford, UK) under contract 17/WM/0333. Informed consent was obtained from each donor before blood collection.

Note that full information on the approval of the study protocol must also be provided in the manuscript.

Field-specific reporting

Please select the one below that is the best fit for your research. If you are not sure, read the appropriate sections before making your selection.

[x] Life sciences [ ] Behavioural & social sciences [ ] Ecological, evolutionary & environmental sciences

For a reference copy of the document with all sections, see nature.com/documents/nr-reporting-summary-flat.pdf

Life sciences study design

All studies must disclose on these points even when the disclosure is negative.

Sample size	Sample size was determined by pilot studies. All sample sizes are indicated in figure legends.
Data exclusions	No data exclusions were made
Replication	All data were reproduced with a minimum of 3 independent experiments, or where human primary cells were used, with a minimum of three different donors. Where representative data are shown as western blots for mucin shedding, the quantified data from replicate experiments are included within the figure and repeat blots are available in the source data files.
Randomization	Individual cell lines were compared within experiments and did not require randomization. Human samples were from donors anonymized at source, donor grouping into experimental groups was random, and covariate analysis was not relevant to the experimental design.
Blinding	No blinding was done in this study as the operators performing the analysis were also involved in collecting and labeling samples. However all within-experiment sample groups were run at the same time so prior knowledge had no impact on data output.

Reporting for specific materials, systems and methods

We require information from authors about some types of materials, experimental systems and methods used in many studies. Here, indicate whether each material, system or method listed is relevant to your study. If you are not sure if a list item applies to your research, read the appropriate section before selecting a response.

Materials & experimental systems

n/a	Involved in the study	
	x	Antibodies
	x	Eukaryotic cell lines
x		Palaeontology and archaeology
x		Animals and other organisms
x		Clinical data
x		Dual use research of concern
x		Plants

Methods

n/a	Involved in the study	
x		ChIP-seq
	x	Flow cytometry
x		MRI-based neuroimaging

Antibodies

Antibodies used	Flow cytometry<br>Anti-human CD43-APC clone L10 (Invitrogen 10746863)<br>Anti-CD43-APC clone DFT-1 (Becton-Coulter B49195)<br>Anti-CD43-PE clone DFT-1 (Becton-Coulter A32560)<br>Anti-CD43-AF647 clone DFT-1 (Santa-Cruz sc6256)<br>Anti-human CD45-PE (MEM-28, Abcam ab134202)<br>Anti-human MUC1-PE clone 16A (Biolegend 355604)<br>Anti-human MUC24-PE clone 67D2 (Biolegend 324808)<br>Anti-human PSGL-1-PE clone TC2 (Invitrogen MA1-10117)<br>Anti-human ADAM10-BV421 clone 11G2 (BD Biosciences 742787)<br>Anti-human CD4-Percp-Cy5.5 clone RAPA-T4 (Biolegend, 300530)<br>Anti-human CD8 clone SK1 on AF700 (Biolegend 344724)<br>Anti-human active caspase-3 antibody clone Asp175 (Cell Signalling Technologies 9661)<br>Anti-human CD71 clone OKT9-FITC (Invitrogen 14-0719-82)<br>Anti-human CD3 clone clone UCHT1-V450 (BD Bioscience 560366)<br>Anti-Myc clone 9E10 (Santa Cruz SC-40)<br>Anti-rabbit IgG (H+L) Alexa Fluor 647, (Invitrogen, A21245)<br>Anti-rabbit Alexa Fluor 546 (Invitrogen, A11010)<br><br>Western blotting<br>Anti-human ADAM10 polyclonal (Merck AB19026)<br>Anti-human PSGL-1 clone 108 (Thermo Fisher MA5-29555)<br>Anti-human MUC1 clone MH1-CT2 (ThermoFisher MA5-11202) at 1/500<br>Anti-human CD43 clone SP55 (Invitrogen MA5-16339)<br>Anti-human CD45 clone EP322Y (Abcam Ab40763)<br>Anti-rabbit HRP (BioRad 1706505)<br>Anti-Armenian hamster-HRP (ThermoFisher PA1-32045)
Validation	Antibodies against CD43, PSGL-1, MUC-1 and ADAM10 were screened against their respective wild-type and knock-out cell lines for appropriate reactivity. In addition, all commercially-sourced antibodies were validated by the manufacturers and validation data are provided with each reagents or accessible at the product website. The manufacturers tested each antibody by immunofluorescent staining analyzed by flow cytometry, immunofluorescent microscopy, or western blot analysis.

Eukaryotic cell lines

Policy information about cell lines and Sex and Gender in Research

Cell line source(s)	Acute T cell lymphoid leukemia (T-ALL) line CEM (ATCC CCL-119) and its CD43-KO counterpart (CD43KO) were kindly provided by the Ardman lab.<br>Acute T cell lymphoid leukemia (T-ALL) lines Jurkat E6.1 (ATCC CRL-2063) and HPBALL (ATCC CRL-2629), and the pre-B-ALL line NALM6 (ATCC CRL-3273) and pro-monocytic line U937 (ATCC CRL-1593.2) were obtained from the Sir William Dunn School of Pathology cell bank.<br>HEK 293T cells (ATCC 293T - CRL-3216) were obtained from the Sir William Dunn School of Pathology cell bank.
Authentication	The cell lines were not authenticated
Mycoplasma contamination	The cell lines were tested negative for mycoplasma
Commonly misidentified lines<br>(See ICLAC register)	No commonly misidentified cell lines were used in this study

Flow Cytometry

Plots

Confirm that:

- [x] The axis labels state the marker and fluorochrome used (e.g. CD4-FITC).
- [x] The axis scales are clearly visible. Include numbers along axes only for bottom left plot of group (a 'group' is an analysis of identical markers).
- [x] All plots are contour plots with outliers or pseudocolor plots.
- [x] A numerical value for number of cells or percentage (with statistics) is provided.

Methodology

Sample preparation	For analysis of cell phenotype in the absence of, or during apoptosis, cell lines or monocytic monocyte-depleted PBMCs or purified primary CD4+ T cells were centrifuged for 5 min at 400 g. Pellets were resuspended in 100 µL cold annexin V binding buffer (BD Pharmingen) for 20 min at 4°C in the dark with annexin V-FITC or -pacific blue (1:100, Biolegend), near-IR fixable viability dye (1:1000, Invitrogen) and fluorophore-conjugated primary antibodies. All labelling was done in conjunction with the corresponding concentration-matched isotype control antibody. After labelling, cells were washed with cold annexin V binding buffer where appropriate, centrifuged for 2 min at 400 g at 4°C, and fixed with 4% paraformaldehyde (Sigma Aldrich) for 10 min at RT. Cells were washed with PBS and permeabilized with perm buffer (Biolegend), and in some experiments labelled with anti-human active caspase-3 antibody (Asp175, Cell Signalling Technologies) at 1:400. After 30 min incubation at 4°C, cells were washed with FACS wash buffer and incubated with appropriate secondary antibodies where required for 30 min at 4°C.
Instrument	After washing, cells were analyzed by flow cytometry using a Cytoflex LX flow cytometer (Beckman Coulter)
Software	Data were processed using the FlowJo-V10 software (FlowJo, LLC) analyzed using Graphpad Prism V9, and formatted using Adobe Illustrator.
Cell population abundance	N/A
Gating strategy	Isotype controls and Fluorescence Minus One (FMO) controls were performed for all colors to gate on positive and negative populations. Gating on the relevant cell population was set according to Forward Scatter (FSC) and Side Scatter (SSC) before doublet and live/dead cell dye (Thermo Fisher) to allow exclusion of dead cells with dye-permeable membranes. Where monocyte-depleted PBMC samples were used, gates were set to differentiate CD4+ and CD8+ T cell subsets. Subsequent gating for all samples was carried out on annexin V and/or activated caspase-3 labelling to differentiate apoptotic from non-apoptotic cells within the same total cell population.

- [x] Tick this box to confirm that a figure exemplifying the gating strategy is provided in the Supplementary Information.
