# Supplementary figures and images for: Apoptosis-mediated ADAM10 activation removes a mucin barrier promoting T cell efferocytosis
Source: Nat Commun. 2024 Jan 15;15:541. doi: 10.1038/s41467-023-44619-8 (PMC10789802; doi:10.1038/s41467-023-44619-8)

Source Data for Figure  
S9d

**b**

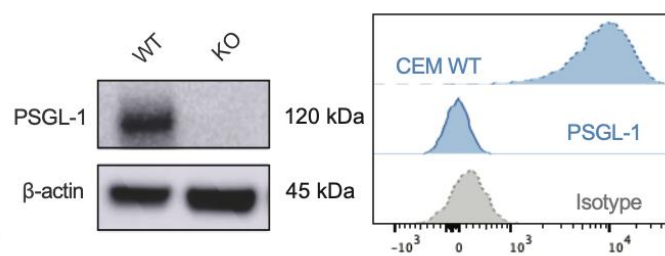

PSGL-1

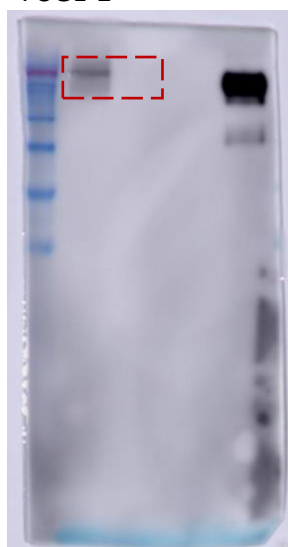

B-actin

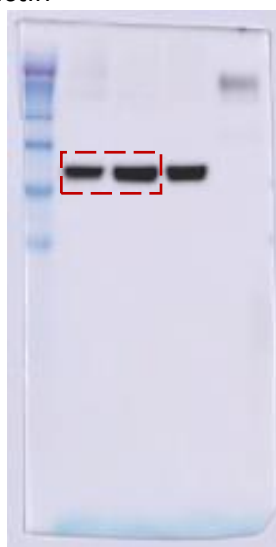

Source Data for Figure  
S9e

**C**

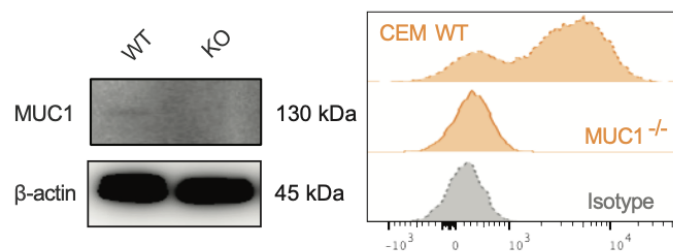

MUC1

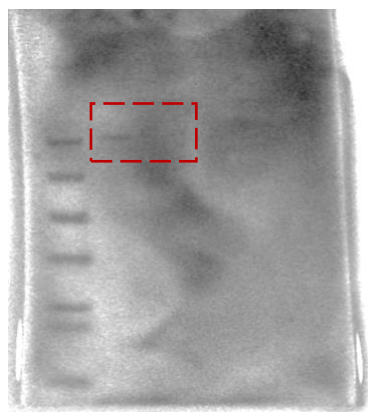

B-actin

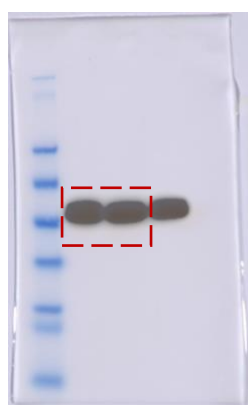

Supplement: Supplementary file 4 — Source Data [file 41467_2023_44619_MOESM4_ESM.zip › 20231129 Source Data for upload 1.12.23/Source Data FigS9d, e.pdf]

Source Data for  
Figure 2i

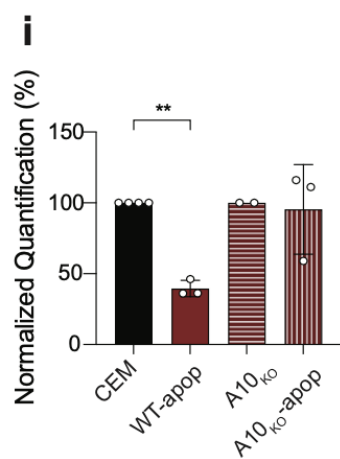

CD43 staining: CEM, WT-apop, A10<sub>KO</sub>, A10<sub>KO</sub>-apop

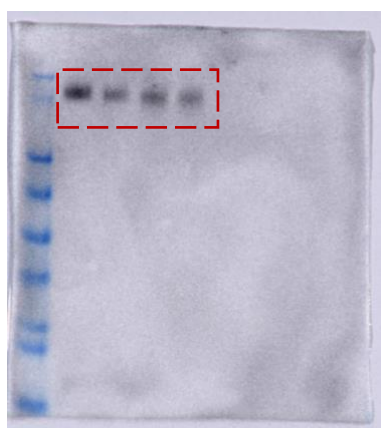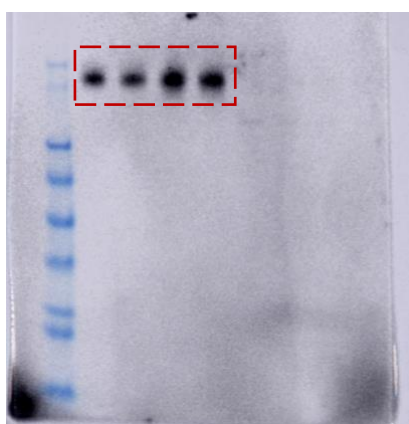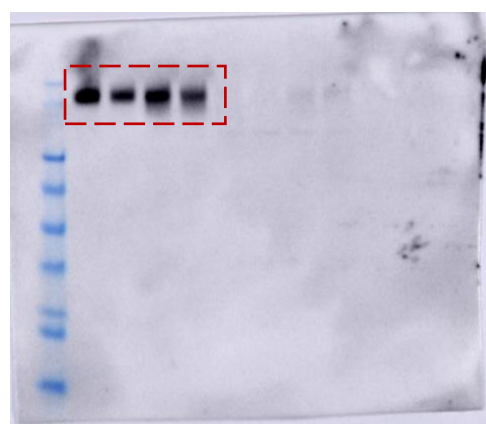

b-actin:

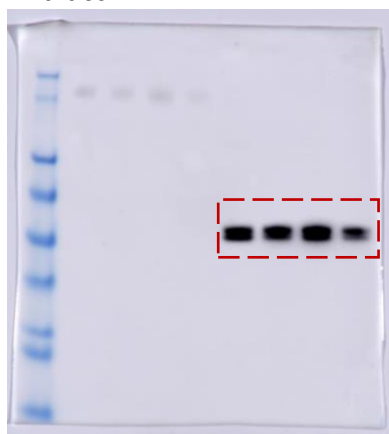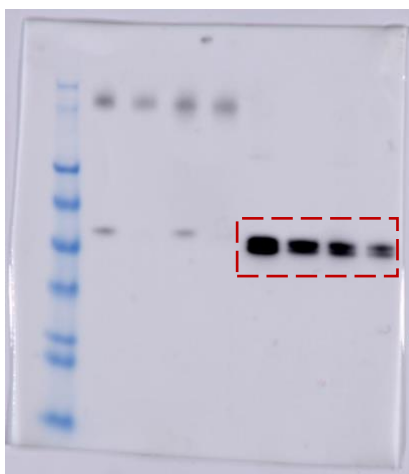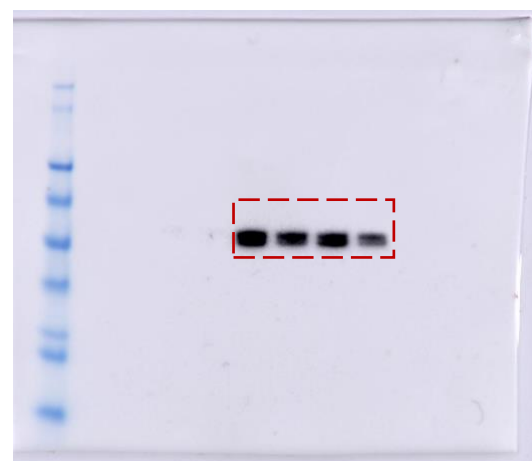

Supplement: Supplementary file 4 — Source Data [file 41467_2023_44619_MOESM4_ESM.zip › 20231129 Source Data for upload 1.12.23/Source Data Fig2i.pdf]

Source Data for  
Figure 2h

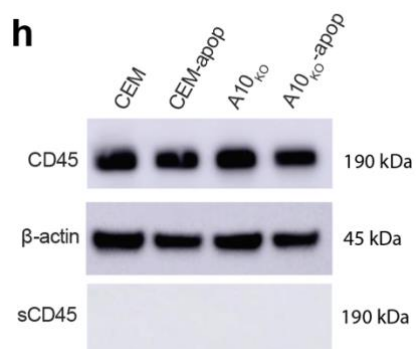

IB: CD45

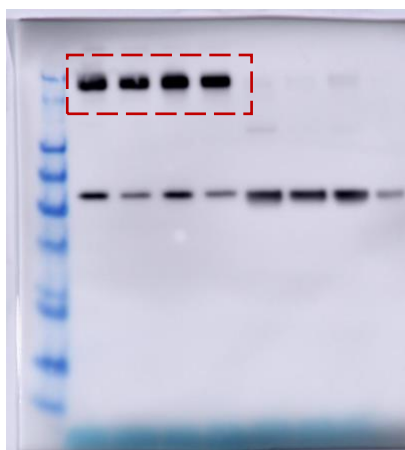

IB: B-Actin

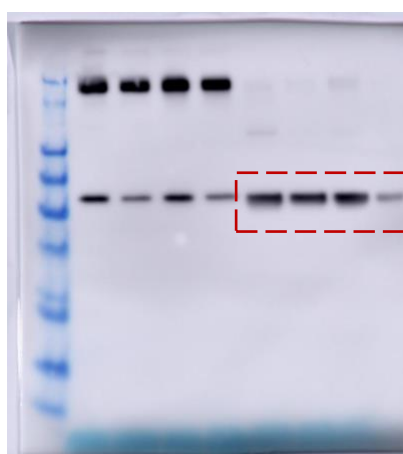

IB: sCD45

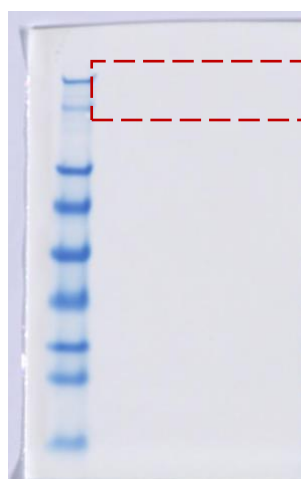

Other sCD45 blots not shown:

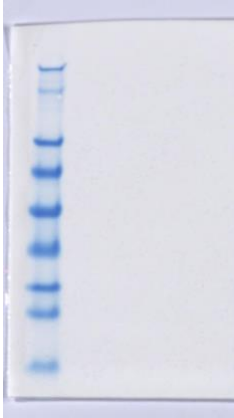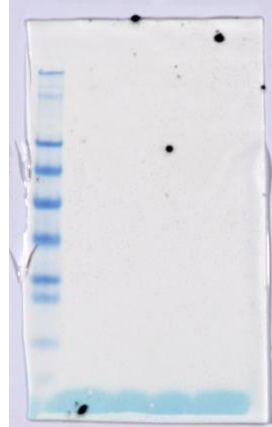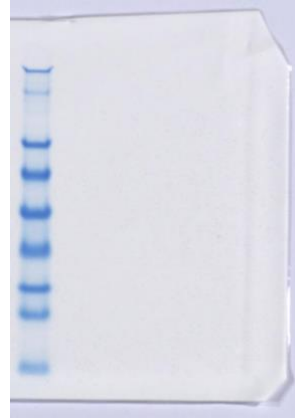

Supplement: Supplementary file 4 — Source Data [file 41467_2023_44619_MOESM4_ESM.zip › 20231129 Source Data for upload 1.12.23/Source Data Fig2h.pdf]

Source Data for  
Figure 2j

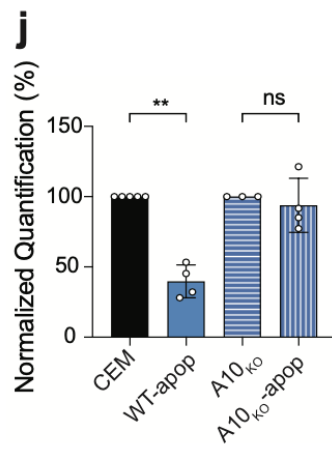

PSGL-1 staining: CEM, WT-apop, A10<sub>KO</sub>, A10<sub>KO</sub>-apop

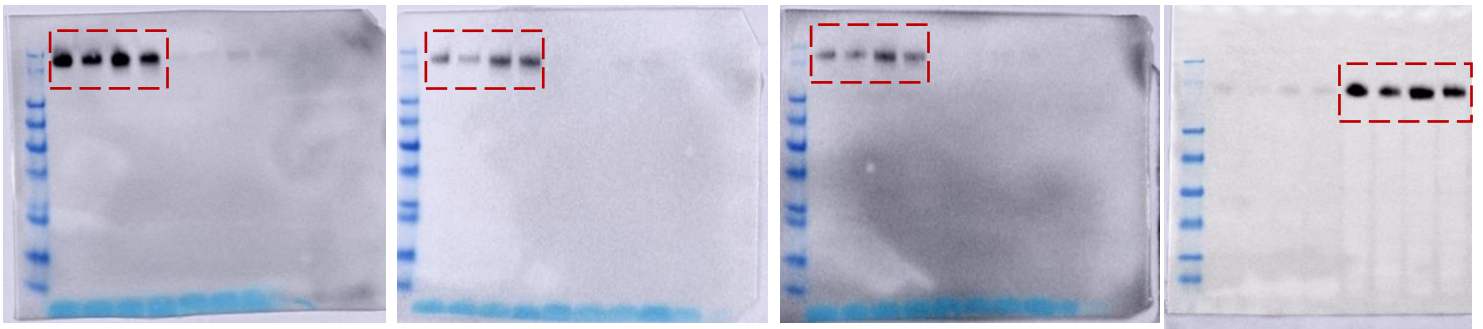

B-Actin

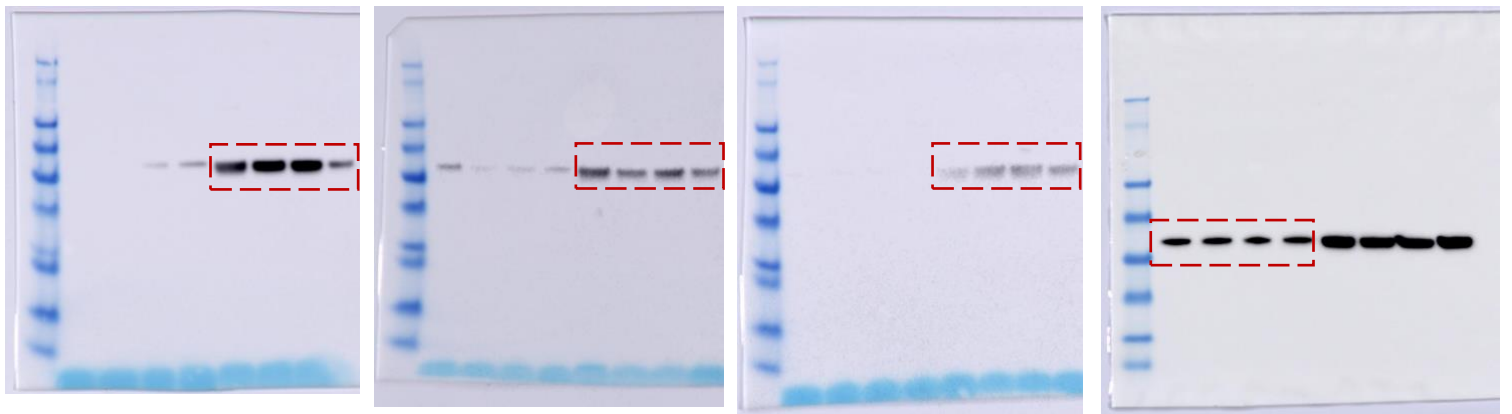

Supplement: Supplementary file 4 — Source Data [file 41467_2023_44619_MOESM4_ESM.zip › 20231129 Source Data for upload 1.12.23/Source Data Fig2j.pdf]

Source Data for  
Figure 2k

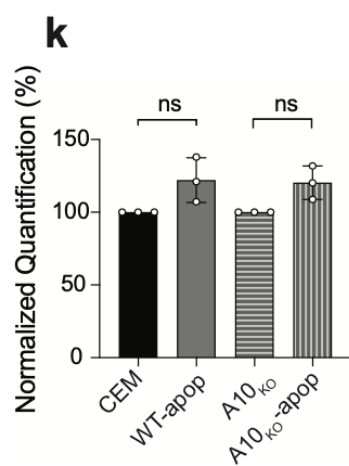

CD45 staining: CEM, WT-apop, A10<sub>KO</sub>, A10<sub>KO</sub>-apop

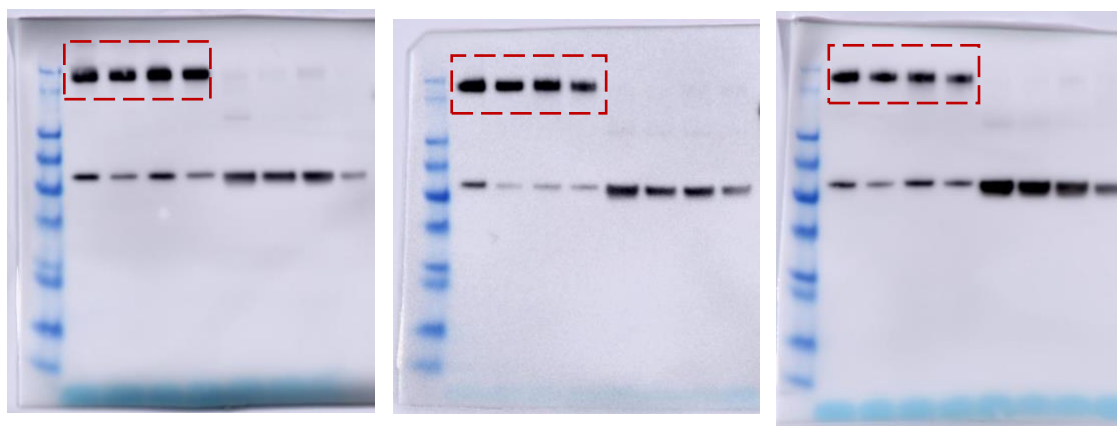

B-Actin

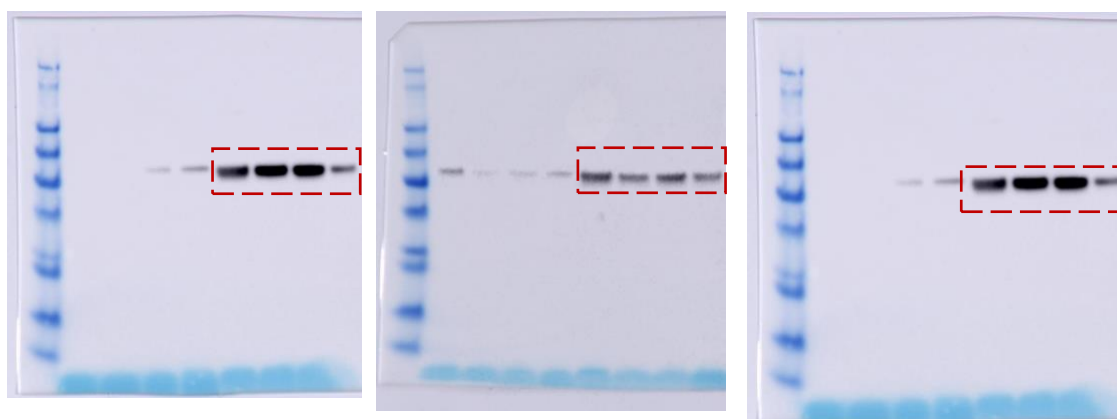

Supplement: Supplementary file 4 — Source Data [file 41467_2023_44619_MOESM4_ESM.zip › 20231129 Source Data for upload 1.12.23/Source Data Fig2k.pdf]

Source Data for Figure  
S9b

**a**

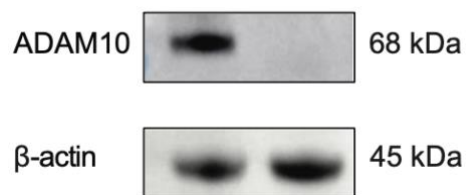

ADAM10

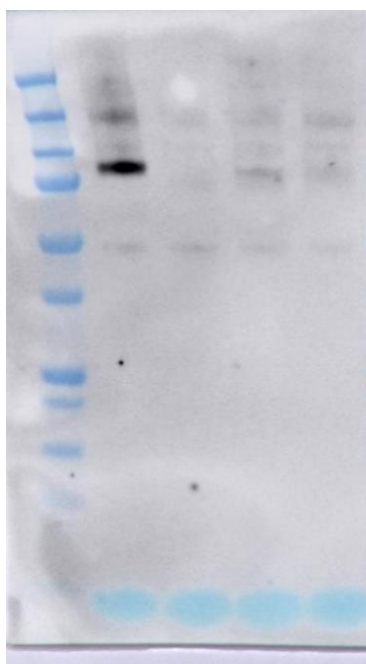

B-Actin

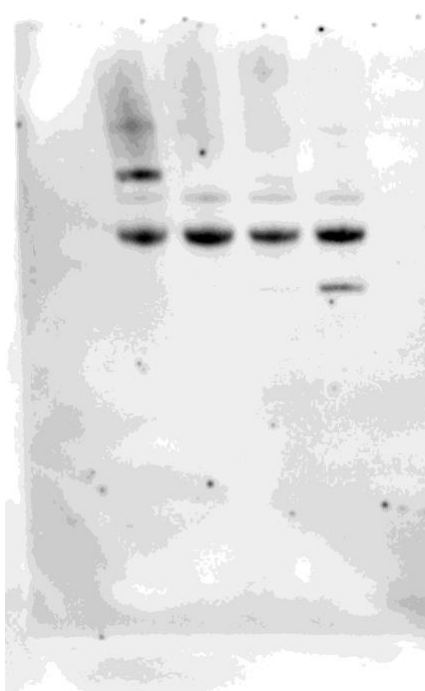

Supplement: Supplementary file 4 — Source Data [file 41467_2023_44619_MOESM4_ESM.zip › 20231129 Source Data for upload 1.12.23/Source Data FigS5a.pdf]

Source data for  
Supplementary Figure 5d

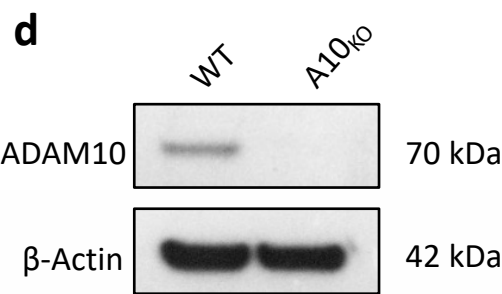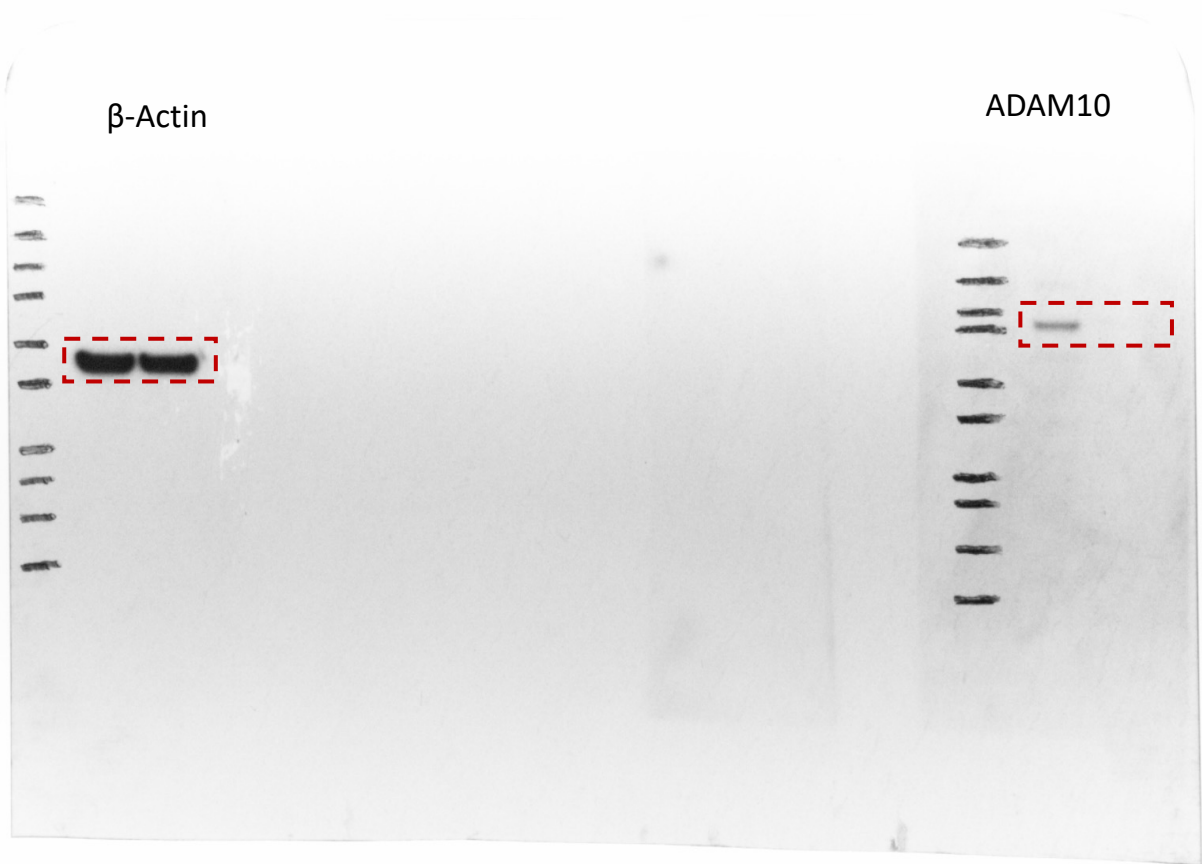

Supplement: Supplementary file 4 — Source Data [file 41467_2023_44619_MOESM4_ESM.zip › 20231129 Source Data for upload 1.12.23/Source data FigS5d.pdf]

Source Data  
Figure 2f

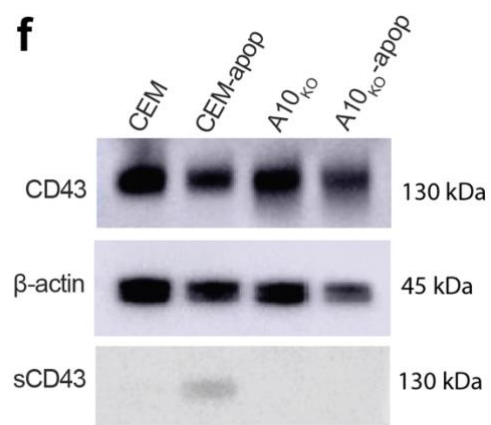

IB: CD43

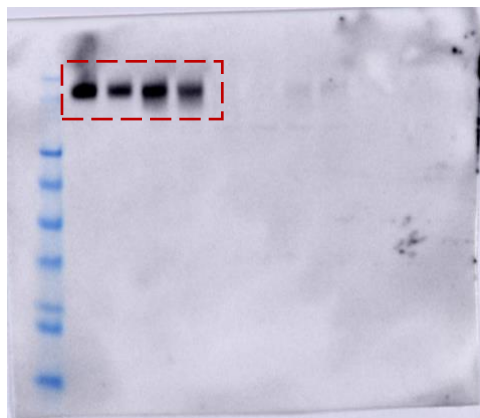

IB: B-Actin

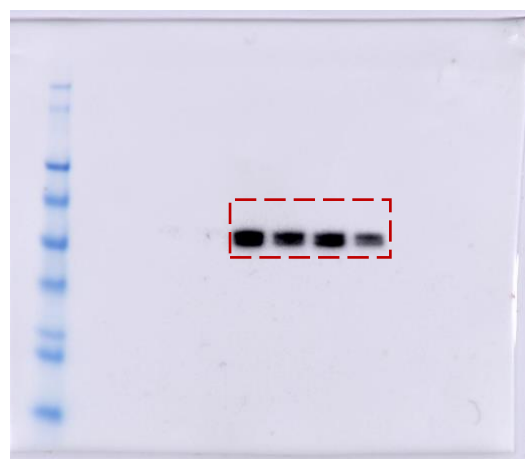

IB: sCD43

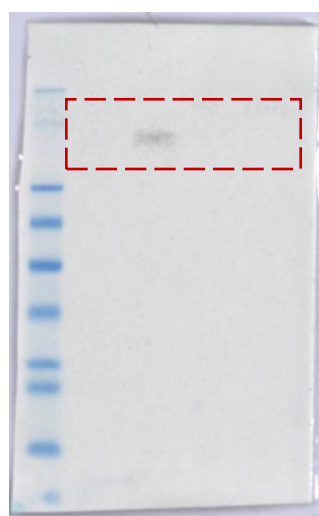

Supernatant western blot repeats for sCD43:

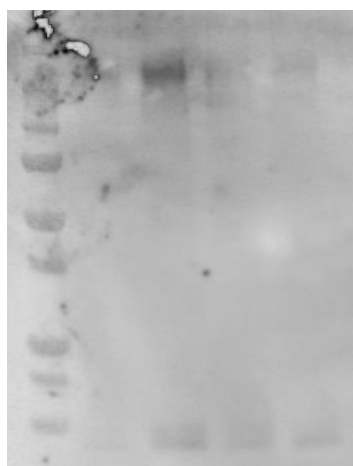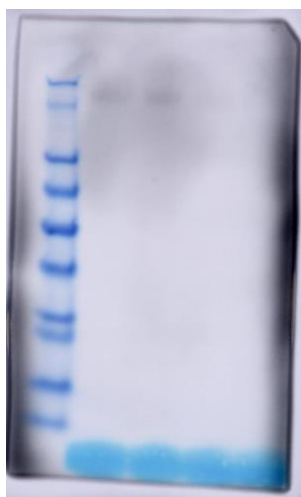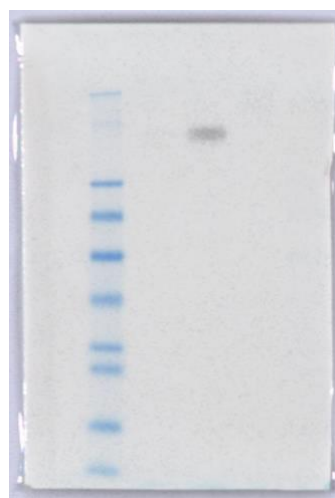

Supplement: Supplementary file 4 — Source Data [file 41467_2023_44619_MOESM4_ESM.zip › 20231129 Source Data for upload 1.12.23/Source Data Fig2f.pdf]

Source Data for  
Figure 2g

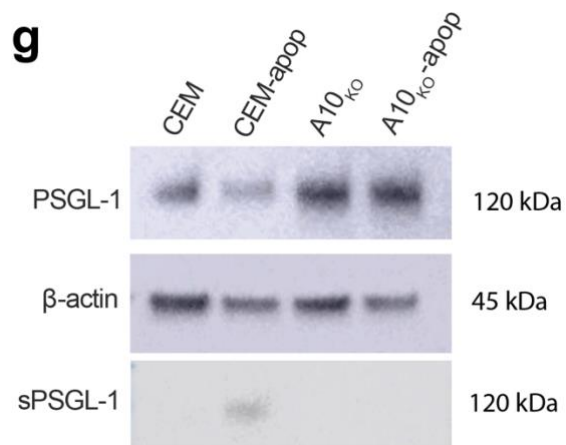

IB: PSGL-1

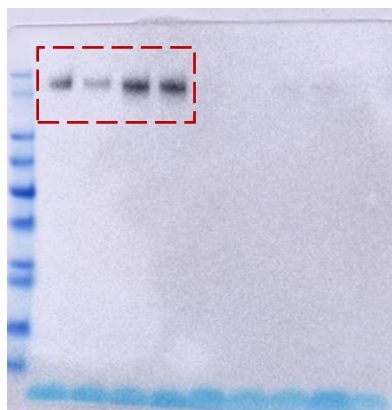

IB: B-Actin

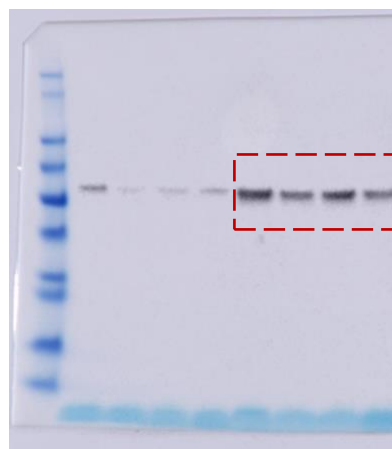

IB: sPSGL-1

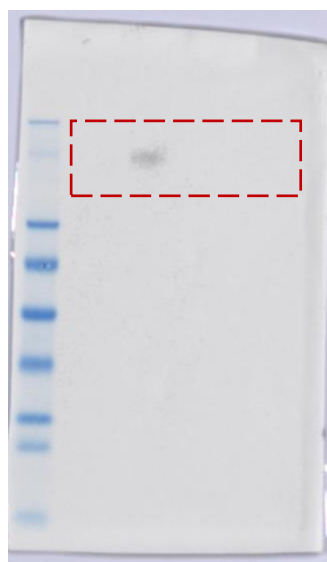

Other sPSGL-1 repeats not shown:

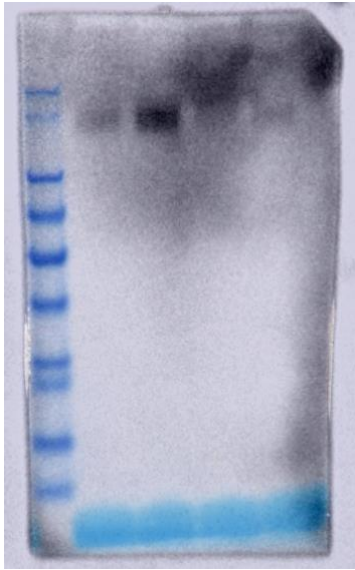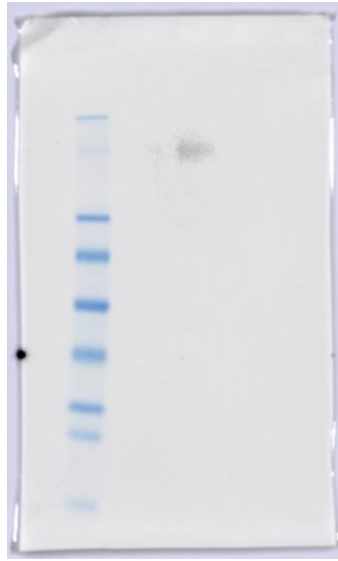

Supplement: Supplementary file 4 — Source Data [file 41467_2023_44619_MOESM4_ESM.zip › 20231129 Source Data for upload 1.12.23/Source Data Fig2g.pdf]
